# Characteristic Distribution of Ciguatoxins in the Edible Parts of a Grouper, *Variola louti*

**DOI:** 10.3390/toxins13030218

**Published:** 2021-03-17

**Authors:** Naomasa Oshiro, Hiroya Nagasawa, Kyoko Kuniyoshi, Naoki Kobayashi, Yoshiko Sugita-Konishi, Hiroshi Asakura, Takeshi Yasumoto

**Affiliations:** 1 National Institute of Health Sciences, 3-25-26 Tonomachi, Kawasaki, Kanagawa 210-9501, Japan; h1r08a.n@gmail.com (H.N.); k-kuniyoshi@nihs.go.jp (K.K.); hasakura@nihs.go.jp (H.A.); 2 Department of Food and Life Science, School of Life and Environmental Science, Azabu University, 1-17-71 Fuchinobe, Chuo-ku, Sagamihara, Kanagawa 252-5201, Japan; n-kobayashi@azabu-u.ac.jp (N.K.); yoshikoni2020@gmail.com (Y.S.-K.); 3 Tama Laboratory, Japan Food Research Laboratories, 6-11-10 Nagayama, Tama, Tokyo 206-0025, Japan; yasumotot@jfrl.or.jp

**Keywords:** ciguatera, ciguatoxin, LC-MS/MS, CTX1B, 52-*epi*-54-deoxyCTX1B, 54-deoxyCTX1B

## Abstract

Ciguatera fish poisoning (CFP) is one of the most frequently encountered seafood poisoning syndromes; it is caused by the consumption of marine finfish contaminated with ciguatoxins (CTXs). The majority of CFP cases result from eating fish flesh, but a traditional belief exists among people that the head and viscera are more toxic and should be avoided. Unlike the viscera, scientific data to support the legendary high toxicity of the head is scarce. We prepared tissue samples from the fillet, head, and eyes taken from five yellow-edged lyretail (*Variola louti*) individuals sourced from Okinawa, Japan, and analyzed the CTXs by LC-MS/MS. Three CTXs, namely, CTX1B, 52-*epi*-54-deoxyCTX1B, and 54-deoxyCTX1B, were confirmed in similar proportions. The toxins were distributed nearly evenly in the flesh, prepared separately from the fillet and head. Within the same individual specimen, the flesh in the fillet and the flesh from the head, tested separately, had the same level and composition of toxins. We, therefore, conclude that flesh samples for LC-MS/MS analysis can be taken from any part of the body. However, the tissue surrounding the eyeball displayed CTX levels two to four times higher than those of the flesh. The present study is the first to provide scientific data demonstrating the high toxicity of the eyes.

## 1. Introduction

Ciguatera fish poisoning (CFP) is one of the most frequently encountered seafood poisoning syndromes; it is caused by the consumption of the marine finfish contaminated with ciguatoxins (CTXs) [1,2,3,4]. CTXs bind to receptor-site 5 of the alpha subunit of the voltage-gated sodium channel and cause hyperexcitability of the nerve membrane [5]. CFP is associated with gastrointestinal, cardiovascular, and neurological disorders, and more than 170 symptoms and signs have been reported [1]. The patients recover within a few days in mild cases, but the symptoms last for months or years in severe cases [5]. CFP mostly occurs in tropical and subtropical regions of the Pacific, the Indian Ocean, and the Caribbean Sea and is commonly reported in the South Pacific islands, including French Polynesia, Fiji, Cook Island, and Kiribati, among others [4,6]. In recent years, there have been a series of reports of CFP in areas where it does not typically occur. For example, cases were recorded in the Canary (Spain) and Madeira (Portugal) Islands, which belong to Macaronesia in northwestern Africa in the eastern Atlantic Ocean, in 2004, and occurrences have continued since then [7,8]. Therefore, there are concerns about the expansion of CFP endemic areas. In Japan, Okinawa and the Amami Islands are located in the subtropical region; several cases of CFP are reported annually, though at much lower numbers than in the South Pacific Islands [9,10,11]. From 1989 to 2011, 78 CFP events were officially reported to the Government of Japan, 90% (70 events) of which were in Okinawa. The most implicated fish was the yellow-edged lyretail *Variola louti* (16 events, 21%), followed by one-spot snapper *Lutjanus monostigma* (12 events, 15%), and red snapper *Lutjanus bohar* (11 events, 14%) [12].

**Figure 1:**
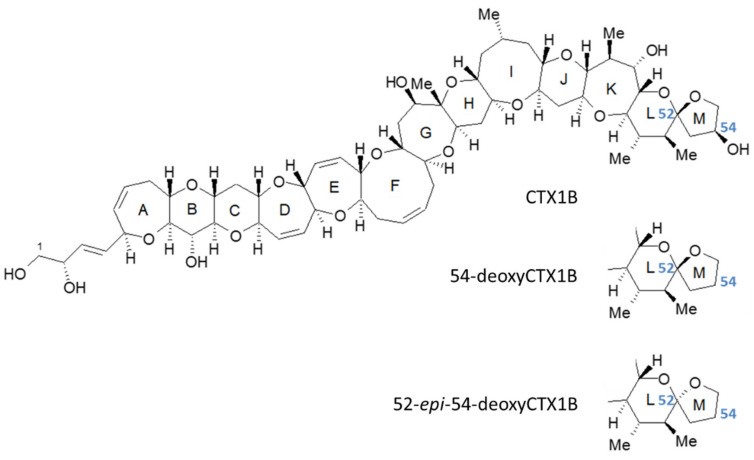
Structures representative of ciguatoxin-1B (CTX1B), 52-*epi*-54-deoxyCTX1B, and 54-deoxyCTX1B, implicated in CFP in Okinawa, Japan.

CTXs are produced by some species from the dinoflagellate genera *Gambierdiscus* and *Fukuyoa* and transmitted to herbivorous animals and carnivorous fish via the food chain [13,14]. CTXs are naturally occurring macromolecules consisting of ladder-shaped polyether, with more than 20 analogs reported from the Pacific Ocean and classified into CTX1B-type and CTX3C-type toxins based on their skeletal structure [15]. In our previous studies, we reported the regional and species-specific features of the CTX profiles of fish in the Pacific using liquid chromatography–tandem mass spectrometry (LC-MS/MS) analysis [16,17,18,19]. In the specimens captured in Hawaii, French Polynesia, Minamitorishima (Marcus Island), and mainland Japan, both CTX1B-type and CTX3C-type toxins were detected [16]. In contrast, only CTX1B-type toxins were detected in the Okinawa and Amami regions’ specimens. In *V. louti* captured from Okinawan waters, only three CTX1B analogs (CTX1B, 52-*epi*-54deoxyCTX1B and 54-deoxyCTX1B; Figure 1), were present and the relative levels of these three analogs were comparable.

The majority of CFP cases arise from consumption of the fish fillet, but among the inhabitants of tropical islands, it is believed that the head and viscera are more toxic than the fillet [4,9]. Additionally, there have been reports that the symptoms are more severe among patients who consumed the head and/or the viscera than in those who consumed only the flesh [4,20,21,22,23,24]. Usually, the whole or half-cut head, without removing the eyes, is broiled, steamed, stewed, deep-fried, or souped. The legends of Okinawa, Japan, point out the high risks of eating the heads, and the actual number of incidents was reported in an early epidemiological study in Okinawa [25,26]. Thus, avoiding the consumption of the viscera and the head is recommended [4,27]. The presence of high levels of CTXs in the liver of ciguateric fish is well documented [26,28,29,30], providing the basis for the use of the livers as the source of toxin isolation [31,32]. Conversely, toxicity data on the CTX levels in head samples are scarce [29].

**Figure 2:**
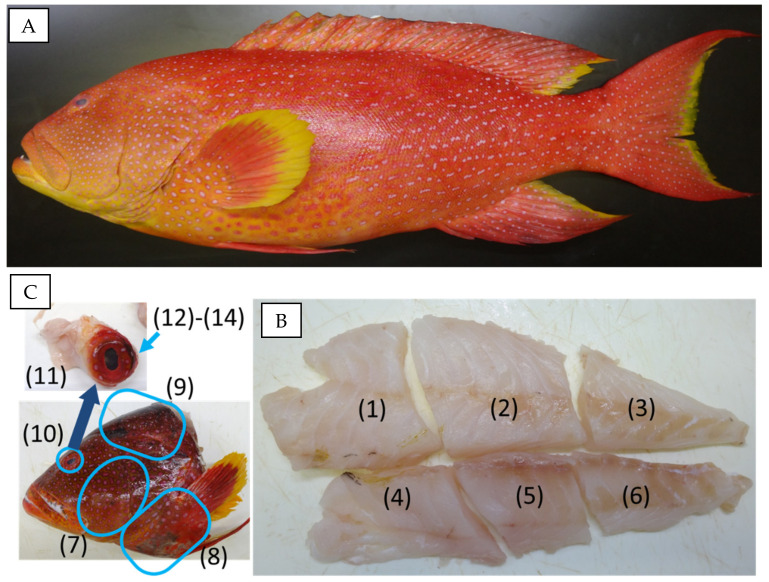
A specimen (D, ID 160135) of *V. louti* used for this study (**A**) and the locations of samples used for LC-MS/MS analyses. (**B**) The half-body fillet was divided into six parts (#1–#6, bottom right). (**C**) From the head, the flesh samples taken from the cheek (#7), the collar (#8), and the cavalry (#9); the eyeball (#10) and the tissue surrounding eyeball (#11). The eyeball was further separated into samples #12–#14, as shown in Table 1.

In this study, we analyze CTXs by LC-MS/MS to disclose their levels and profiles in the flesh samples dissected from the fillet and head (cheek, collar, and cavalry) and the eye samples, the edible parts of fish mentioned in the legends. We used five individuals of the carnivorous fish *V. louti*, the yellow-edged lyretail (Figure 2A), one of the representative species of CFP in Okinawa, Japan. The presence of CTX1B, 52-*epi*-54deoxyCTX1B, and 54-deoxyCTX1B in these specimens was confirmed by preliminary analysis.

## 2. Results

Six and three flesh samples were prepared from the fillet (#1–#6) and head (#7–#9), respectively, as shown in Figure 2B,C. Eye samples (#10), composed of the eyeball and the surrounding tissue (#11), were taken, and the eyeballs were further separated from Specimens B–E (#12–#14), as shown in Table 1.

**Figure 3:**
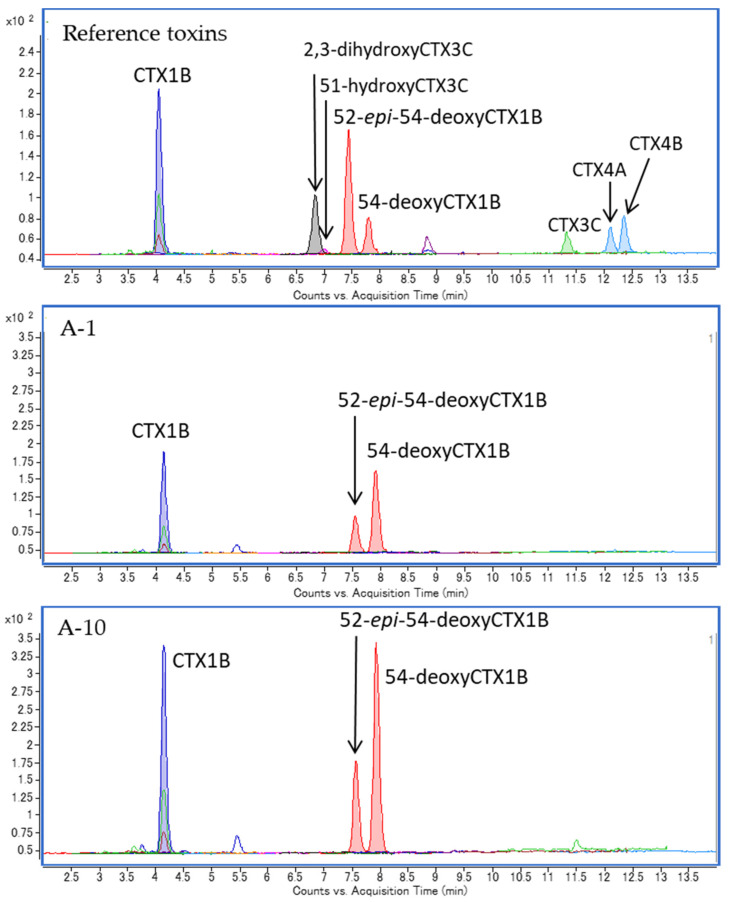
LC-MS/MS chromatograms of reference toxin mixture (top), flesh (A-1, middle), and eye (A-10, bottom) samples of Specimen A.

**Figure 4:**
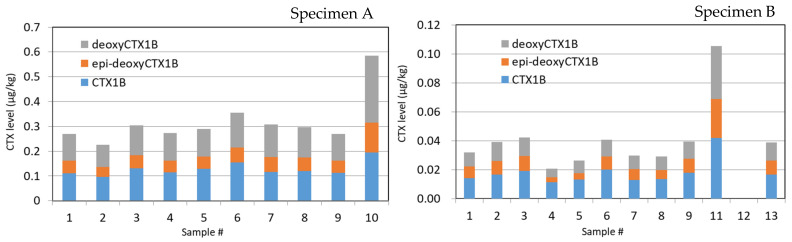

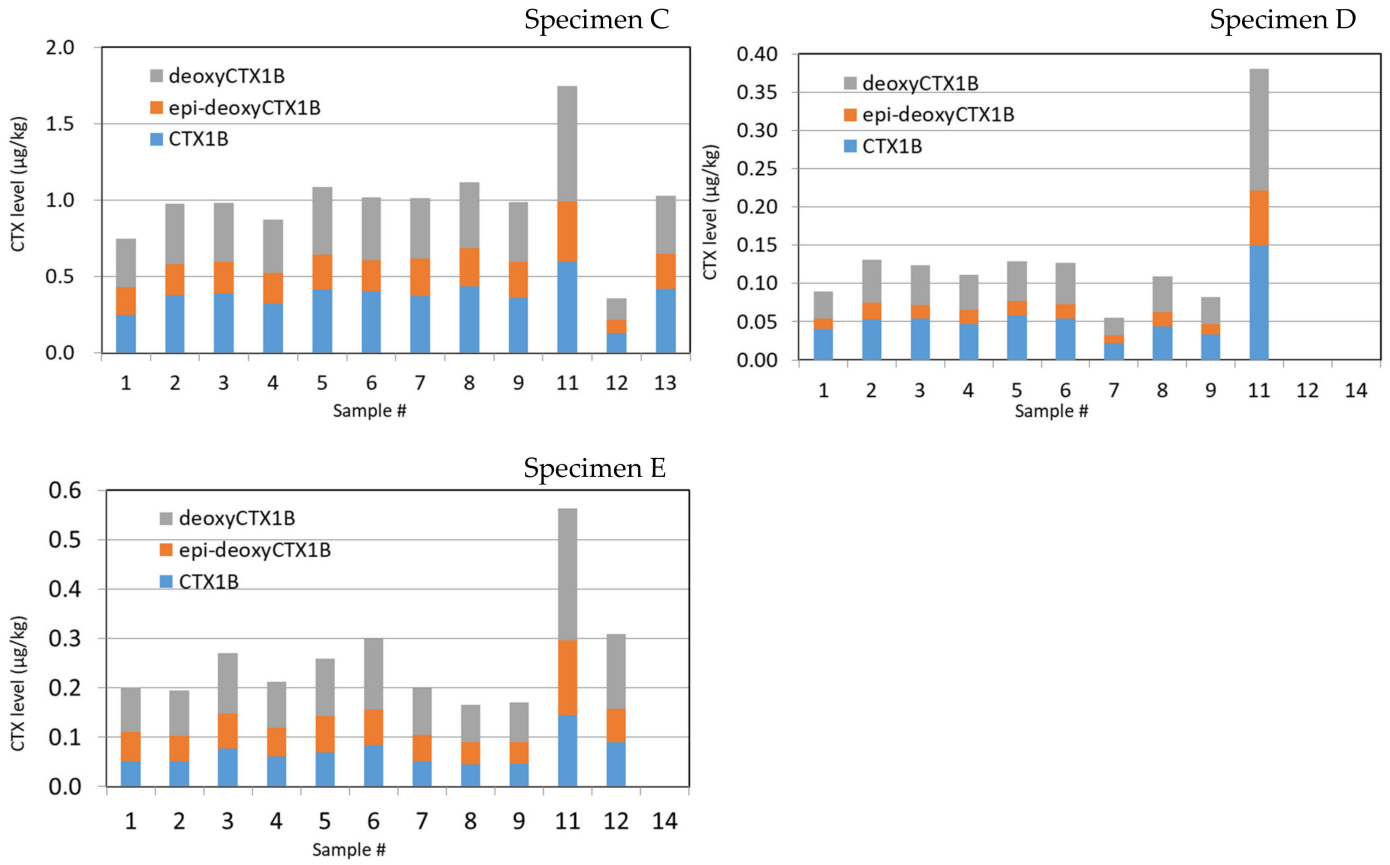
The levels of CTX1B analogs detected in samples prepared from Specimens A–E.

Three CTX1B-type analogs, including CTX1B, 52-*epi*-54-deoxyCTX1B, and 54-deoxyCTX1B, were detected in all specimens. However, neither the fourth CTX1B-type nor the CTX3C-type analog was detected in any samples (Figure 3; Figure 4). Detection of only three CTX1B analogs is characteristic in the carnivorous fish from Okinawa [16,17]. Representative chromatograms are shown in Figure 3. The ratios of the three CTX1B analogs were similar within all specimens (Figure 4).

### 2.1. Specimen A

The total CTX levels (average ± SD (standard deviation)) in the flesh samples from the filet (#1–#6) and the head (#7–#9) were similar, measuring 0.287 ± 0.043 and 0.292 ± 0.012 μg/kg, respectively (Table 2 and Figure 4). The eye sample (#10), comprised of the eyeball and the surrounding tissue, was mixed, and LC-MS/MS analysis was applied. The level of CTXs was 0.585 μg/kg, twice as high as that of the flesh samples (Table 3). The ratios of three CTX analogs within the specimens were similar (Figure 4).

### 2.2. Specimen B

The CTX levels in the flesh samples were 0.033 ± 0.009 μg/kg in the filet (#1–#6) and 0.033 ± 0.006 μg/kg in the head (Table 2). To clarify the source of the high CTX levels in the eye sample, as observed in Specimen A, the eye sample was further separated into three portions in Specimen B: the surrounding tissue (#11), the inner contents of the eyeball, including the lens (#12), and the outer membrane of the eyeball (#13). The highest levels of CTX1B analogs were detected in #11, with 0.106 μg/kg, which was three times higher than of the flesh samples (#1–#9). The level in #13 was 0.039 μg/kg, and in #12, it was less than the limit of detection (LOD, <0.001 µg/kg) (Table 3).

### 2.3. Specimens C

The samples were prepared in the same way as those of Specimen B. The levels in the filet, the head, and samples #11–#13 were 0.948 ± 0.120, 1.039 ± 0.068, 1.747, 0.360, and 1.031 μg/kg, respectively (Table 2; Table 3). While the detected levels in Specimen C (0.360–1.747 μg/kg) were much higher than in Specimen B (0.021–0.106 μg/kg), the relative levels in respective sample portions were similar (Table 3).

### 2.4. Specimens D

CTXs were detected at the levels of 0.118 ± 0.016 and 0.082 ± 0.027 μg/kg in the filet and the head, respectively (Table 2). We prepared separate tissue samples, consisting of the surrounding tissue (#11), inner contents (#12), and lens (#14) from the eye, to locate where the highest levels of CTXs had originated. Among them, CTXs were detected only in #11 (0.380 μg/kg), which exhibited levels 3.2 and 4.6 times higher than in the filet and the head, respectively (Table 3).

### 2.5. Specimen E

Although the levels of CTX1B and 54-deoxyCTX1B in all flesh samples (#1–#9) were similar, the levels of 52-*epi*-54-deoxyCTX1B were much higher in the fillet samples #1–#6 than in #7–#9 taken from the head of Specimen E (Figure 4A, Figure S1). In addition to the different ratios of the three CTX1B analogs in the flesh and head, splitting the peak top of 52-*epi*-54-deoxyCTX1B of samples of the filet made us suspect the presence of interfering substances in these samples. These samples were reanalyzed using a different gradient system (Gradient II), and the peaks of 52-*epi*-54-deoxyCTX1B were separated from those of the interferent, as shown in Figure 4B and Figure S2. The levels in the filet and the head were at 0.239 ± 0.043 and 0.179 ± 0.018 μg/kg, respectively (Table 2).

The eye samples were prepared equally to those of Specimen D (Table 1), and CTXs were detected in #11 and #12 at 0.563 and 0.308 μg/kg, respectively (Table 3). The level in #11 was two to three times higher than of the filet and the head (Table 3). No CTXs were detected in #14. While no CTXs were detected in #12 of Specimen D, the same analogs were detected at relatively high levels in the corresponding sample of Specimen E (Table 3). The different results between Specimens D and E might be ascribable to the cross-contamination of surrounding tissues.

## 3. Discussion

According to the inhabitants of the tropical islands where CFP is prevalent, the head and viscera are more likely to cause CFP than the fillet. In addition, many papers reported that the patients who consumed these portions displayed the most severe symptoms among individuals who had shared the same fish [4,21,22,23]. In order to obtain evidence upholding this legend, we prepared samples from the fillet and the head, the edible parts of the fish, and separately analyzed CTXs by LC-MS/MS. The gills, scales, and viscera were not tested in this study because they are not considered edible tissues. We separated the fillet further into small six sections, depending on the distances from the head and tail. The fillet from the belly side is believed to be fatty compared to the dorsal side. For the head, we took the flesh from the cheek, collar, and calvary. CTX levels across the specimens and the levels and profiles in the flesh samples were similar across the specimens. The toxin contents and toxin profiles in these small sections are important—first, to prevent food poisoning, and secondly, for the accurate evaluation of the fish under testing. In the case of beef, the variations of quality among the body parts are well understood. No comparable data exist as to the variation in fish or the profiles of toxins. Much attention has been paid to the accuracy of the analytical method to quantify CTXs. If CTXs are unevenly distributed in the fish body, the validity of the analytical data will be governed by which part the sample has been taken from. The present study demonstrates that flesh samples for toxin analysis can be arbitrarily taken from all parts of the body of *V. louti* except the eye areas. This finding is important for designing the protocol for testing fish for various purposes, including food safety, business, legal actions, and more.

**Figure 5:**
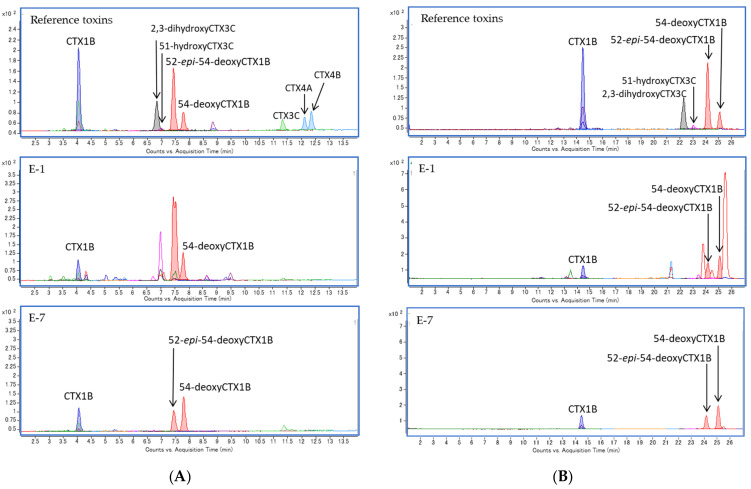
LC-MS/MS chromatograms of reference toxin mixture (top) and flesh samples of the fillet (E-1, middle) and cheek (E-7, bottom) of Specimen E. The gradient systems used were conditions Gradient I (**A**) and II (**B**).

In Specimen E, interference peaks were detected in the fillet samples but not in the flesh from the head or the tissues of the eye (Figure 5). The presence of the interferences only in the filet is interesting, and further investigation to uncover the structural information of these substances will be carried out.

In the samples prepared from the eyes, namely, the tissue surrounding the eyeball, CTXs were detected at two to four times higher levels than in the flesh samples. This tissue is known to contain high contents of polyunsaturated fatty acids, docosahexaenoic acid (DHA) and icosapentaenoic acid (eicosapentaenoic acid (EPA)) [33,34,35]. The high levels of CTXs in the eyes will be an interesting phenomenon for future studies. Although high in concentration, the eyes alone cannot account for the legends of higher toxicity of the head. According to Li et al. [29], CTXs were consistently detected in various tissues of grouper *Epinephelus coioides* over 30 days of exposure. CTX levels ranked from high to low in the following order: liver, intestine, gill, skin, brain, and muscle. The brain and other cerebrum tissues in the head were suspected to be rich in CTXs because of the abundance of the voltage-dependent sodium channels [5,36]. However, the difficulty of separation and the small quantity available for analysis prevented us from analyzing CTXs from those tissues in this study. Attempts will be made in the future to test the cerebrum tissues in the head and the backbone. Finally, the reliability and high efficacy of LC-MS/MS analysis in studying food safety and the body distribution of CTXs have been demonstrated.

## 4. Conclusions

The levels and profiles of the CTXs (CTX1B, 52-*epi*-54-deoxyCTX1B, and 54-deoxyCTX1B) in tissues were shown to be comparable among samples prepared from the same individual. This even distribution was revealed by LC-MS/MS analyses using the fillet and head and eye samples of five specimens of a representative ciguateric fish, *Variola louti.* Any part of the flesh, except for the eyes, can be used for analyses to judge the safety of the fish or to produce ecological data. The tissues of the eyes were found, for the first time, to contain CTX levels two or four times higher than that of the flesh.

## 5. Materials and Methods

### 5.1. Reference Toxins and Reagents

The reference toxin mixture was comprised of eight CTX analogs, including CTX1B, 52-*epi*-54-deoxyCTX1B, 54-deoxyCTX1B, CTX4A, CTX4B, 2,3-dihydroxyCTX3C, 51-hydroxyCTX3C, and CTX3C, all of which were pure or near-pure toxins prepared from natural sources and identified by spectroscopic analysis [15,37,38,39,40,41]. The levels of the detected toxins were quantified using the reference toxins Ciguatoxin-1B (43.3 ± 1.3 ng) and 52-*epi*-54-deoxyCTX1B (58.4 ± 2.5 ng). Since the reference material for 54-deoxyCTX1B was unavailable, its levels were quantified using 52-*epi*-54-deoxyCTX1B.

Acetone, hexane, and ethyl acetate of Primepure grade, diethyl ether of Guaranteed reagent grade, and methanol and acetonitrile of liquid chromatography–mass spectrometry (LC-MS) grade were purchased from Kanto Chemical Co., Inc. (Tokyo, Japan). Ammonium formate solution (1 mol/L) and formic acid were of high-performance liquid chromatography (HPLC) grade (Wako Chemical Industry, Ltd., Osaka, Japan). Ultra-pure water was supplied by the Milli-Q^®^ Integral Water Purification System for Chemical Analysis (Millipore, Bedford, MA, USA).

### 5.2. Fish Specimens

The five individuals of *V. louti* used were found to be contaminated with CTX1B, 52-*epi*-54-deoxyCTX1B, and 54-deoxyCTX1B and selected from our ciguateric fish collection, purchased in Okinawa, Japan (Specimens A–E, Table 1, Figure 2A). The head and a fillet were taken from the body. The fillet was cut into 6 parts (#1–#6), as shown in Figure 2B. The following four samples were prepared from the heads: the flesh from the cheek (#7), the collar (#8), and cavalry (#9), and the eyeball combined with surrounding tissue (#10), as shown in Figure 2C. Each eye sample was separated, as shown in Table 1. In Specimen A, the whole part was mixed (#10). Since the CTX level of the eye sample (#10) was the highest within the samples (#1–#10) of Specimen A, and in order to explore the highly contaminated portion in the eye, eye samples (#10) in Specimens B and C were separated into the surrounding tissue (#11), inner contents including the lens (#12), and outer membrane (#13). In Specimens D and F, to confirm the existence of high-level CTXs in the surrounding tissue and to verify whether the CTXs are present in the eyeball’s inner contents or lens, samples #11, #12, and #14 were prepared and analyzed.

### 5.3. Extraction and Sample Preparation for LC-MS/MS

Extraction and preparation of the fish flesh followed the method in previous studies (Figure S3) [18,19]. Flesh (5 g) was extracted with acetone (15 mL) twice, and the extracts were combined and evaporated to remove the acetone. The remaining aqueous extracts were partitioned with diethyl ether (5 mL) twice, and the combined diethyl ether layer was dried completely and dissolved in 90% methanol (*v/v*, 1.5 mL). The aqueous methanol was defatted with hexane (3 mL, twice), and the remaining solution was dried completely to obtain the crude extract.

The crude extract was dissolved in ethyl acetate-methanol (9:1, *v/v*, 5 mL) and applied to a Florisil column (500 mg, GL Sciences Inc., Tokyo, Japan) that had been preconditioned with the same solvent. The eluent, containing nonabsorbed substances, was collected and dried under a nitrogen stream. The residue was dissolved with acetonitrile (5 mL) and applied to a primary and secondary amine cartridge column (PSA, 200 mg, GL Sciences Inc., Tokyo, Japan), preconditioned with methanol and acetonitrile. CTXs were eluted first with acetonitrile and then methanol (3 mL). Both acetonitrile and methanol eluates were dried under a nitrogen stream and dissolved in methanol to be analyzed by LC-MS/MS. A 1-mL portion of the solution was equivalent to 5 g of the flesh.

The preparation of the eye samples (#10–#14) followed that of the flesh samples. When the sample was smaller than 5 g, the whole sample was used, and the extraction and pretreatment procedures were carried out without any change. The sample solution used for LC-MS/MS was adjusted to 5 g tissue-equivalent/mL.

### 5.4. LC-MS/MS Analysis

LC-MS/MS analysis was carried out with the Agilent 1290 HPLC system coupled with an Agilent 6460 Triple Quadrupole MS instrument (Santa Clara, CA, USA) (Table S1). Briefly, 5 µL of sample solution was injected into a Zorbax Eclipse Plus C18 column (2.1 × 50 mm id, 1.8 μm, Agilent Technologies, Santa Clara, CA, USA), at 40 °C. Eluate A was water containing 5 mM ammonium formate and 0.1% formic acid. and Eluate B was methanol. The gradient system (Gradient I) was as follows: 0.0–0.25 min (60% B); 0.25–0.50 min (60–75% B); 0.50–12.0 min (75% B); 12.0–14.0 min (90% B); 14.1–20 min (100% B). When the presence of an interfering substance was suspected, the sample was reanalyzed using another gradient system. The gradient condition for Gradient II was as follows: 0–0.25 min (50% B); 0.25–0.5 min (50–65% B); 0.5–25 min (65–80% B); 25–27 min (80% B); 27–33 min (100% B). The flow rate was 0.4 mL/min. The target toxins were ionized with electron spray ionization (ESI) equipped with Agilent Jet Stream, and positive ions were monitored with multiple reaction monitoring (MRM) mode. Since [M + Na]^+^ ions were stable and gave no fragment ions, [M + Na]^+^ of each analog was set for both precursor and product ions ([M + Na]^+^ > [M + Na]^+^), with high collision energy to achieve sensitive analysis. The optimized MS parameters were dry gas N_2_, 300 °C, 10 L/min; nebulizer gas N_2_, 50 psi; sheath gas N_2_, 380 °C, 11 L/min; capillary voltage 5000 V; fragmentor voltage 300 V; collision gas N_2_, collision energy 40 eV. The limit of detection (LOD) and the limit of quantitation (LOQ) of CTX1B, 52-*epi*-54-deoxyCTX1B, and 54-deoxyCTX1B were 0.001 and 0.005 µg/kg, respectively.

## Figures and Tables

**Table 1:** Sample preparation from the eyeball and the surrounding tissue taken from the fish specimens.

Specimen	ID ^1^	SL ^2^ (mm)	Weight (g)	Eye Sample Preparation ^3^
A	160024	490	2934	whole parts were mixed (#10)
B	160085	410	1734	surrounding tissue (#11), inner contents, including a lens (#12) and the outer membrane of the eyeball (#13)
C	163077	456	2719	surrounding tissue (#11), inner contents, including a lens (#12) and the outer membrane of the eyeball (#13)
D	160135	490	2594	surrounding tissue (#11), inner contents of the eyeball (#12) and lens (#14)
E	160136	450	2430	surrounding tissue (#11), inner contents of the eyeball (#12) and lens (#14)

^1^ Specimen ID at the National Institute of Health Sciences. ^2^ SL: standard length; ^3^ preparation of the sample from the eyeball and surrounding tissue for LC-MS/MS analysis. Numbers in brackets are the sample codes.

**Table 2:** CTX levels in flesh prepared from the fillet (#1–#6) and head (#7–#9).

Specimen	CTXs Levels (μg/kg) in the Fillet ^1^								CTXs Levels (μg/kg) in the Head ^1^				
	#1	#2	#3	#4	#5	#6	Average	SD ^2^	#7	#8	#9	Average	SD
A	0.270	0.226	0.305	0.274	0.290	0.355	0.287	0.043	0.308	0.297	0.270	0.292	0.019
B	0.032	0.039	0.042	0.021	0.026	0.041	0.033	0.009	0.030	0.029	0.039	0.033	0.006
C	0.749	0.978	0.984	0.873	1.088	1.018	0.948	0.120	1.011	1.117	0.989	1.039	0.068
D	0.089	0.131	0.124	0.111	0.129	0.126	0.118	0.016	0.055	0.110	0.082	0.082	0.027
E	0.201	0.195	0.270	0.212	0.259	0.300	0.239	0.043	0.200	0.166	0.171	0.179	0.018

^1^ The sum of the levels of CTX1B, 52-*epi*-54deoxyCTX1B, and 54-deoxyCTX1B. ^2^ SD: standard deviation.

**Table 3:** CTXs levels in flesh (fillet and head) and eye samples.

Specimen	CTXs Levels (μg/kg) ^1^						
	Fillet ^2^	Head ^3^	#10	#11	#12	#13	#14
A	0.287	0.292	0.585	- ^4^	-	-	-
B	0.033	0.033	-	0.106	<LOD ^5^	0.039	-
C	0.948	1.039	-	1.747	0.360	1.031	-
D	0.118	0.082	-	0.380	<LOD	-	<LOD
E	0.239	0.179	-	0.563	0.308	-	<LOD

^1^ The sum of the levels of CTX1B, 52-*epi*-54deoxyCTX1B, and 54-deoxyCTX1B. ^2^ Average of samples #1-#6. ^3^ Average of samples #7–#9. ^4^ -: not analyzed. ^5^ <LOD: less than the limit of detection (0.001 µg/kg).

## Data Availability

The data presented in this study are available on request from the corresponding author.

## Supplementary Materials

The following materials are available online at https://www.mdpi.com/2072-6651/13/3/218/s1. Figure S1: LC-MS/MS chromatograms in Gradient I of samples (#1–#9, #11, #12, and #14) prepared from Specimen E (ID 160136). Figure S2: LC-MS/MS chromatograms in Gradient II of samples (#1–#9, #11, #12, and #14) prepared from Specimen E (ID 160136). Figure S3: Sample preparation from fish flesh for LC-MS/MS. Table S1: LC-MS/MS conditions for CTX analysis.
