# Supplementary material for: Characteristic Distribution of Ciguatoxins in the Edible Parts of a Grouper, Variola louti
Source: Toxins (Basel). 2021 Mar 17;13(3):218. doi: 10.3390/toxins13030218 (PMC8002984; doi:10.3390/toxins13030218)
Supplement: Supplementary file 1 [file toxins-13-00218-s001.pdf]

Supplementary Materials: Characteristic Distribution of Ciguatoxins in the Edible Parts of a Grouper, *Variola louti*

Naomasa Oshiro, Hiroya Nagasawa, Kyoko Kuniyoshi, Naoki Kobayashi, Yoshiko Sugita-Konishi, Hiroshi Asakura and Takeshi Yasumoto

Table S1. LC-MS/MS conditions for CTX analysis.

LC: Agilent 1290 Infinity (Agilent Technologies)
Column: Zorbax Eclipse Plus C18
(1.8 μm, 2.1 × 50 mm, Agilent Technologies)
Column Temp.: 40 °C
Injection volume: 5 μL
Eluent A: 5 mM ammonium formate in 0.1% formic acid
Eluent B: methanol
Flow rate: 0.4 mL/min
Gradient conditions
1 (for general analysis):
0 min (60%B)–0.25 min (60%B)–0.5 min (75%B)–12 min (90%B)–14 min (90%B)–14.1 min (100%B)
2 (for less polar analogs):
0 min (60%B)–0.25 min (60%B)–0.5 min (75%B)–23 min (90%B)–25 min (90%B)–25.1 min (100%B)
3 (for higher polar analogs):
0 min (50%B)–0.25 min (50%B)–0.5 min (65%B)–25 min (80%B)–27 min (80%B)–27.1 min (100%B)

MS: 6460 Triple Quad LC/MS (Agilent Technologies)
Ionization: ESI with Agilent Jet Stream
Nebulizer: N_2, 50 psi
Sheath gas: N_2, 380 °C, 11 L/min
Dry gas: 300 °C, 10 L/min
Capillary voltage: 5000 V
Fragmentor voltage: 300 V
Collision energy: 40 eV
Monitor ions ($[M+Na]^+$ > $[M+Na]^+$)
m/*z* 1045.6: CTX3C and 49-*epi*CTX3C
m/*z* 1061.6: 2-hydroxyCTX3C and 51-hydroxyCTX3C
m/*z* 1079.6: 2,3-dihydroxyCTX3C
m/*z* 1083.6: CTX4A and CTX4B
m/*z* 1095.6: 2,3,51-trihydroxyCTX3C
m/*z* 1117.6: 52-*epi*-54-deoxyCTX1B and 54-deocyCTX1B
m/*z* 1133.5: CTX1B

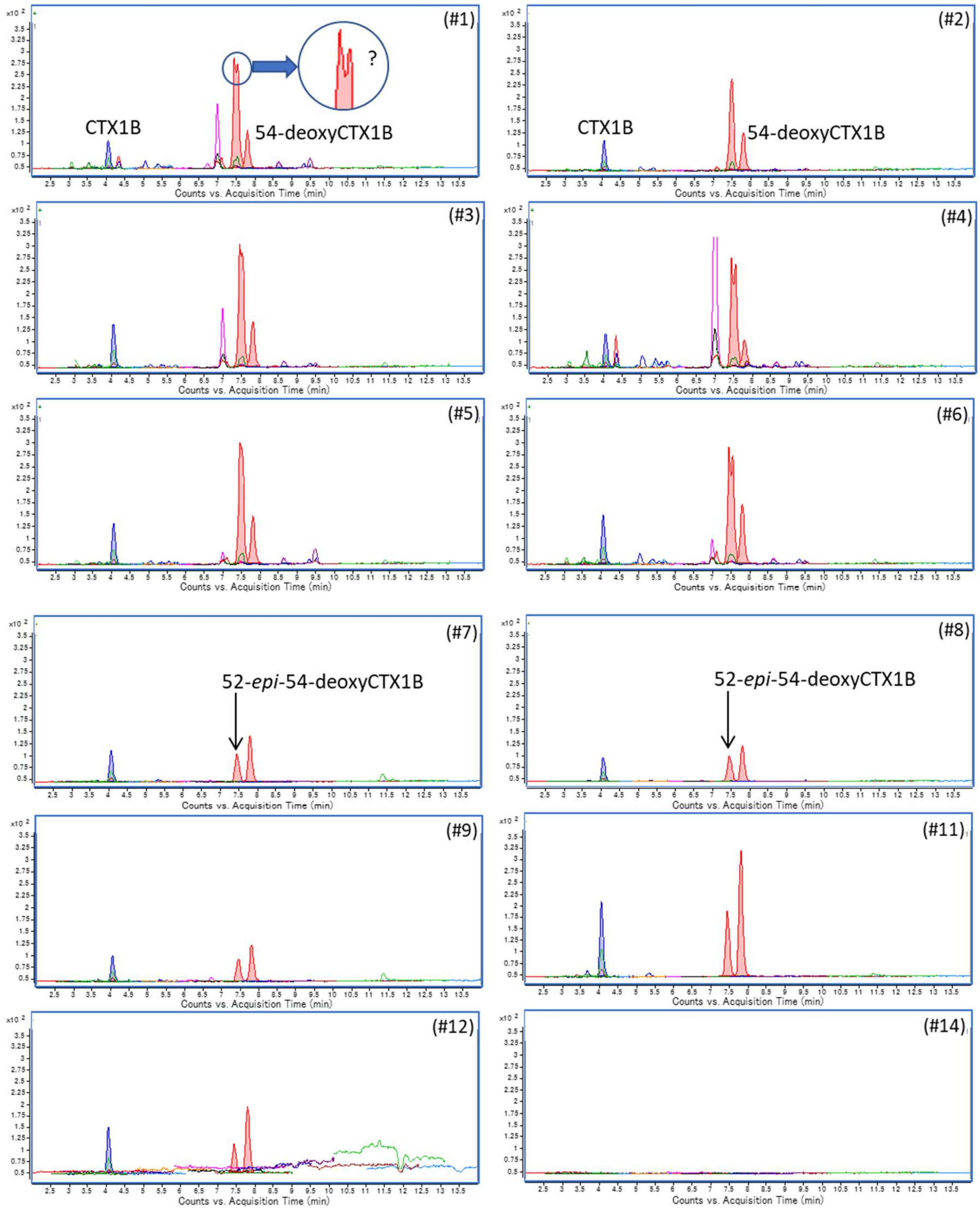

Figure S1. LC-MS/MS chromatograms in Gradient I of samples (#1–#9, #11, #12, and #14) prepared from Specimen E (ID 160136).

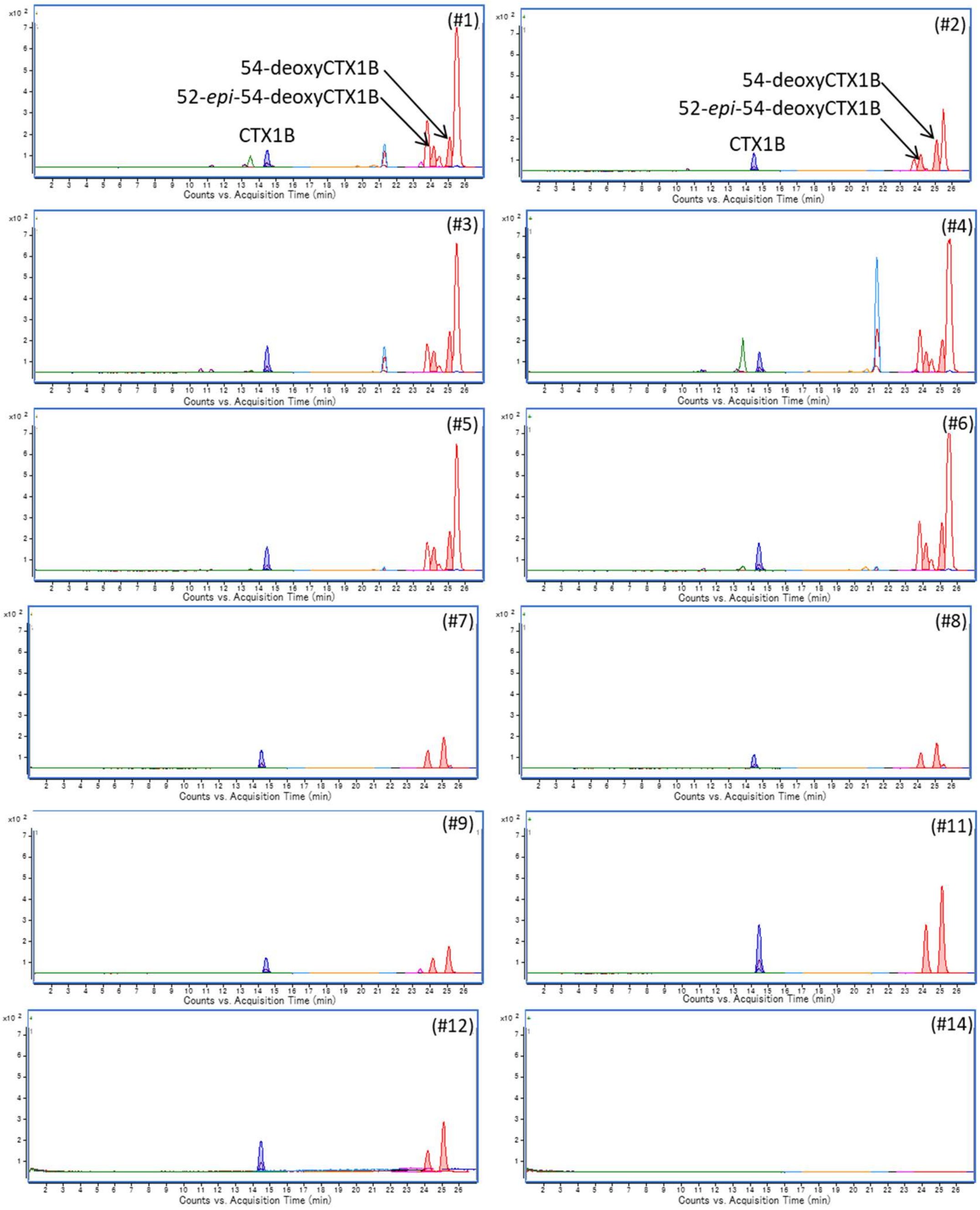

Figure S2. LC-MS/MS chromatograms in Gradient II of samples (#1–#9, #11, #12, and #14) prepared from Specimen E (ID 160136).

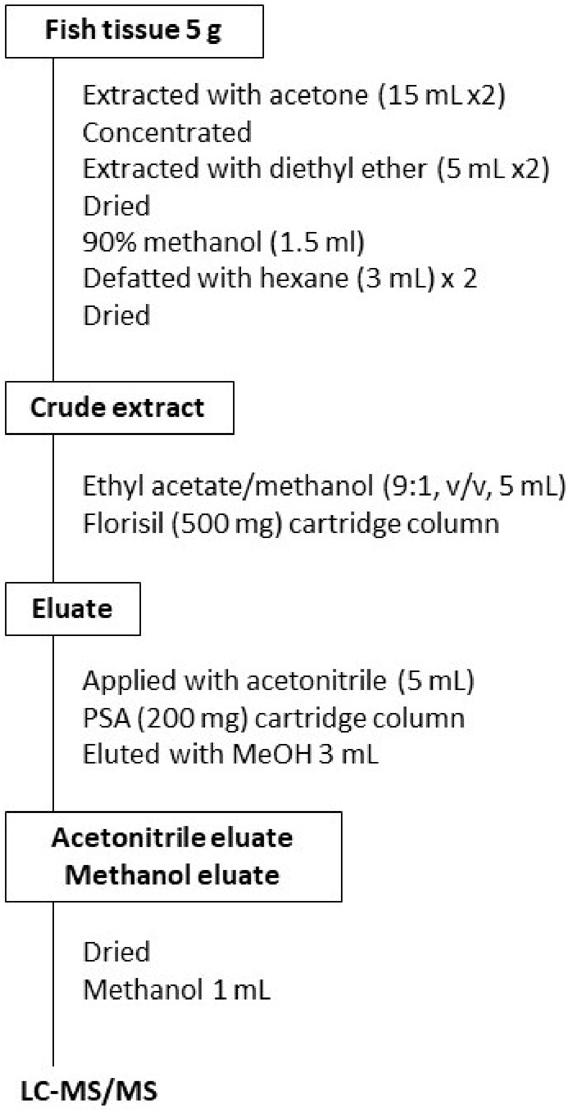

Figure S3. Sample preparation from fish flesh for LC-MS/MS.
